# Contextualising COPD self-management in Malaysia: insights from a qualitative photo-elicitation study of patients-caregiver dyads

**DOI:** 10.7189/jogh.15.04301

**Published:** 2025-11-07

**Authors:** Hani Salim, Abd-Malek Fatin-Syazwani, Natrah Zakaria, Sa’ari Mohamad Yatim, Thanalactchumy Chandrabose, Siti Nurkamilla Ramdzan, Soo Chin Chan, Fadzilah Mohamad, Shariff-Ghazali Sazlina

**Affiliations:** 1 Faculty of Medicine & Health Sciences, Universiti Putra Malaysia, Serdang, Malaysia; 2 Department of Rehabilitation Medicine, Hospital Sultan Idris Shah, Serdang, Malaysia; 3 Faculty of Medicine, Universiti Malaya, Kuala Lumpur, Malaysia; 4 College of Medicine and Veterinary Medicine, Usher Institute, University of Edinburgh, Edinburgh, UK

## Abstract

**Background:**

Chronic obstructive pulmonary disease (COPD) is a leading cause of mortality and morbidity globally, disproportionately affecting low- and middle-income countries (LMICs). Despite pulmonary rehabilitation (PR) being a key intervention, uptake and adherence remain low due to economic, geographical, and sociocultural barriers. We explored the lived experiences of individuals with COPD and their caregivers in Malaysia to identify contextually grounded self-management strategies.

**Methods:**

We employed a qualitative photo-elicitation approach between January and December 2024. We purposively sampled adults with COPD and their caregivers based on age, gender, and ethnicity from a hospital-based outpatient PR centre in Selangor, Malaysia. We conducted semi-structured dyadic interviews at two time points. Participants documented their experiences through photographs, which guided the discussions. Lastly, we transcribed the interviews verbatim and thematically analysed them.

**Results:**

Nine dyads (participant-caregiver pairs) completed two interviews. Participants were men with a mean age of 65.3 (standard deviation (SD) = 3), with GOLD stage 3–4. Caregivers were women, with a mean age of 56.4 (SD = 11). Six dyads identified themselves as Malay ethnicity. Four themes emerged: Navigating economic constraints in COPD self-management, where participants substituted costly devices with low-cost tools (*e.g.* loaded trolleys); Culturally embedded self-management: integrating practices like Qigong and reframing daily chores (*e.g.* folding laundry) as rehabilitation; Technology as a tool for home-based COPD care with participants adapting exercises from internet (*e.g.* Facebook) while caregivers expressed concerns over unverified content; and Family as partners in COPD management, where caregivers not only monitored symptoms but also exercised alongside participants, reporting mutual health benefits. These strategies were seen as essential for sustaining engagement in COPD care.

**Conclusions:**

Photo-elicitation and dyadic interviews revealed how cultural traditions, digital adaptations, and reciprocal caregiving intersect in everyday life, shaping COPD self-management in low-resource settings. Interventions should build on these lived strategies, prioritising context-sensitive, low-cost, and inclusive care models for COPD in LMICs.

Chronic respiratory diseases affect over 544 million people worldwide and are the third leading cause of death, with over 90% of deaths occurring in low- and middle-income countries (LMICs) [1]. Among them, chronic obstructive pulmonary disease (COPD) accounts for 3.3 million deaths annually [2,3], driven by risk factors such as smoking, biomass fuel exposure, and occupational hazards, compounded by limited access to early diagnosis and treatment [3,4]. Despite this, COPD remains under-prioritised in health systems compared to other non-communicable diseases [5].

Global policy frameworks, including the World Health Organization’s Package of Essential Noncommunicable Disease Interventions and ‘Best Buys’, emphasise clinical care and cost-effective interventions but offer limited guidance on integrating culturally congruent practices, digital resourcefulness, or family-inclusive care; critical for sustaining COPD management in LMICs [6,7]. Addressing these gaps is particularly relevant for Malaysia and broader Southeast Asia, where COPD prevalence varies considerably due to differences in awareness, diagnostic capacity, and healthcare infrastructure across urban and rural areas [8].

In Malaysia, COPD-related mortality often results from complications and multimorbidity exacerbated by socioeconomic constraints [9,10]. Financial limitations frequently restrict access to essential services, diagnostics, pharmacological treatments, and pulmonary rehabilitation (PR), an evidence-based intervention known to improve quality of life, functional status, and symptom control [11,12]. However, PR implementation faces notable challenges, including geographic distances, financial barriers, low health literacy, and limited healthcare provider training, all of which contribute to low programme attendance and adherence [13–15].

Addressing these challenges requires a shift toward patient-centred, context-sensitive care models that integrate culturally congruent practices, leverage accessible digital platforms, and mobilise familial caregiving networks. Research indicates that culturally tailored approaches to COPD management, such as incorporating locally recognised practices and mindfulness-based approaches, improve adherence by aligning interventions with patient beliefs and social contexts [16,17]. Digital health tools offer promising, low-cost avenues for self-management, including online education, instructional videos, and peer support via social media [18]. Furthermore, family caregivers play a crucial yet often underacknowledged role in COPD management in LMICs, where formal caregiving support is limited [10,12–14].

Thus, we address the above policy and practice gaps by exploring the lived experiences of individuals with COPD and their caregivers in Malaysia, using an innovative photo-elicitation approach. Photo-elicitation, which uses participant-generated images to prompt discussion, has been shown to elicit richer narratives, enhance recall, and capture contextual and emotional dimensions often overlooked in traditional interviews [19–21]. By examining cultural adaptations, digital resourcefulness, and familial caregiving, we aim to inform the adaptation of PR guidance and COPD policy frameworks for LMIC contexts.

## METHODS

We obtained informed consent from the participant and their caregiver before participation. We followed the Consolidated Criteria for Reporting Qualitative Research to ensure methodological transparency and rigour [22].

### Study design

We employed an interpretive qualitative design using dyadic photo-elicitation, where participants’ own photographs prompted deeper reflection during interviews [21]. We chose this method for its ability to elicit richer narratives and reveal implicit or hard-to-articulate experiences, capturing visual and emotional dimensions often overlooked in conventional interviews [21].

### Setting, recruitment, and sample

We conducted this study between January and December 2024 among people living with COPD and their caregivers. Healthcare professionals identified participants at a hospital-based outpatient PR centre in Selangor, Malaysia. Although recruiting from a single centre may limit generalisability, at the time of the study, this is the only public-funded centre in the state of Selangor that provided a structured PR programme [23]. In this setting, participants are enrolled in an eight-week centre-based PR programme following referral from specialist outpatient clinics or primary care services. We provided the participants with a summary of the centre-based PR programmes (Appendix S1 in the **Online Supplementary Document**).

Eligibility criteria included adults aged ≥18 years with physician-diagnosed COPD of any severity, along with their caregivers, who were attending centre-based PR at the time of the recruitment. Guided by a sampling matrix, we purposively recruited participants to ensure maximum variation in demographic characteristics (*i.e.* age, gender, ethnicity) and COPD severity as defined by the GOLD criteria, which we extracted from PR clinic records of the recent spirometry test. We developed a sampling matrix to guide participant selection, ensuring diversity across key characteristics, including age, gender, ethnicity, and COPD severity. This tool helped monitor recruitment progress and identify underrepresented groups to maximise sample variation [24]. We excluded participants and caregivers who were unable to commit to photo-taking tasks (*i.e.* no access to a camera) and to complete the study stages (*i.e.* the interview at two time points).

A research team member (AF) explained the study procedures, and gave eligible participants and their caregivers up to two weeks to decide on participation before providing written consent. For those who agreed, photo-taking tasks were re-emphasised, and an initial interview was scheduled at a venue convenient to both the participant and their caregiver, without the presence of any non-participants.

### Data collection

We collected sociodemographic information, audio-recorded interviews, and participant-provided photographs. The data collection period lasted 12 months (January to December 2024), during which participants and caregivers completed two interviews at three- to four-month intervals. The first interview took place while participants were still undergoing the PR programme, and the second interview was arranged after they completed it.

### Photo-elicitation interviews

Participants and caregivers were given two to three weeks to capture up to five photographs before each interview using mobile phones or digital cameras. We encouraged them to write brief reflections for each image. Guided photo-taking prompts (Appendix S2 in the **Online Supplementary Document**) elicited narratives around self-management activities, needs, and challenges in COPD, and we later used the photographs in dyadic photo-elicitation interviews [21,25]. Dyadic interviews, conducted with participant-caregiver pairs, enabled simultaneous exploration of perspectives, real-time observation of interactions, and a richer understanding of mutual support and shared experiences often missed in individual interviews. Conducted at two time points and guided by a semi-structured interview guide (Appendix S3 in the **Online Supplementary Document**), the sessions lasted 1–1.5 hours. Participant-provided photographs (with explicit consent) offered contextual and visual insights, while images without consent were discussed but not reproduced. Written consent covered the use of photographs in publications, exhibitions, and non-commercial initiatives. A trained qualitative researcher (HS) facilitated face-to-face interviews in participants’ preferred language, with a note-taker (AF) capturing the flow, dynamics, and non-verbal cues.

### Data handling

We de-identified and assigned unique codes to the interview audio recordings. We reviewed the recordings and began transcription alongside ongoing interviews to refine subsequent discussions. Research team members transcribed all recordings verbatim into local languages, ensuring quality and accuracy through native-speaker checks. Experienced post-doctoral qualitative researchers and bilingual native speakers (HS, SSG, and SNR) translated quotes from the transcripts for report writing into English as needed. Back-translation ensured accuracy, and culturally specific expressions were retained with explanatory footnotes when direct translation was not possible.

### Data analysis

We de-identified, transcribed verbatim and verified the transcript for accuracy. We analysed photographs and transcripts concurrently using reflexive thematic analysis [26], emphasising the team’s interpretive process rather than prescriptive coding steps. The photo-elicitation process shaped the depth and direction of interviews, as photographs often prompted participants to recall details or emotions not easily surfaced through verbal prompts. Images also revealed differences between participants and caregivers, such as when a photo of an online exercise was valued by the participant for convenience but questioned by the caregiver for safety, prompting a richer exploration of these tensions. Visual cues further highlighted subtler aspects of self-management, such as the symbolic meaning of everyday objects (*e.g.* balloons, folded laundry) in maintaining dignity, often overlooked in conventional interviews.

Researchers (HS, AF, SS, and SNR) independently conducted the initial coding, drawing on both textual and visual data. Early discussions centred on what each researcher found striking – for example, how low-cost innovations (*e.g.* balloons, loaded trolleys) went beyond substitutions to represent acts of ingenuity that preserved dignity and independence. We contested some codes – for instance, a photograph of Facebook exercise videos was first coded as digital resourcefulness, but later, following caregiver input, reinterpreted as reflecting safety concerns and trust in unverified information. Similarly, we initially categorised daily chores (*e.g.* folding clothes) as routine activities, but after reviewing photo-narrative pairs, we reframed them as purposeful rehabilitation due to their deliberate use as physical therapy.

Visual analysis focussed on content, composition, and symbolic meanings of photographs, which were triangulated with interview data to enhance analytic depth and credibility. Integrating visual and textual analysis is recommended in photo-elicitation research, as it allows exploration of both explicit and implicit dimensions of experience, offering richer insights than either source alone [19,21]. In some cases, photographs shifted interpretation – for example, an image of a caregiver exercising with the participant was recoded from caregiver support to reciprocal health benefits after both described shared motivation and mutual gains.

We iteratively refined the themes through team discussions, collapsing overlapping codes (*e.g.* cost-saving strategies and resource re-purposing became economic adaptations) and expanding underdeveloped ones (*e.g.* family involvement grew into family as partners in COPD management). We also compared themes across dyads and time points, identifying where perspectives converged (*e.g.* value of cultural practices like Qigong) and diverged (*e.g.* caregiver caution *vs.* participant confidence in online resources).

We considered data saturation, which guided the number of dyads interviewed, reached when no new insights emerged [27]. We observed this after interviewing the eighth pair and confirmed it with the ninth. The research team monitored sufficiency throughout, and thematic repetition across cases indicated that the data set was rich and comprehensive for addressing the study objectives. We used NVivo, version 12 (Lumivero, Denver, Colorado, USA) for data management and analysis.

### Study rigour

To ensure the trustworthiness of this qualitative study, we applied Lincoln and Guba’s (1985, 1989) four criteria [28,29] (Appendix S4 in the **Online Supplementary Document**), which strengthen the quality and reliability of qualitative research [24,30]:

- Credibility: Member checking was conducted, where two participants and their caregiver reviewed a summary of key findings to ensure their experiences were accurately represented. This approach was adopted due to resource limitations and the observation of strong consistency between the summaries and participants’ narratives.
- Transferability: Detailed descriptions of the study setting, participants, and data collection process were provided to allow others to assess whether the findings apply to different contexts.
- Dependability: An audit trail was maintained to document research decisions and analytical steps. Triangulation, involving multiple researchers in data analysis, ensured consistency and reduced individual bias.
- Confirmability: Reflexive journaling (Appendices S4 and S5 in the **Online Supplementary Document**) helped track the main researcher's assumptions, ensuring that findings reflected participants' perspectives.

### Stakeholder engagement

Two COPD patient-caregiver pairs, who were not study participants, contributed to patient and public involvement activities. They ensured study materials and outcomes were relevant, clear, and culturally appropriate, thereby enhancing the acceptability and impact of study recommendations. Beyond refining study materials, the patient and public involvement contributors also reviewed photo prompts and draft interview guides, ensuring their cultural and contextual appropriateness. Their input led to the inclusion of prompts emphasising caregiver support and daily routines, which were critical in surfacing relational dynamics often missed in traditional patient-centred interviews.

At the end of the study, we conducted a stakeholder workshop. Study participants and their caregivers who agreed to take part in this workshop joined clinicians and allied health professionals to share their lived experiences, further informing the co-design of a multilingual, accessible leaflet on understanding COPD and PR. This activity advocated for the routine integration of patient and caregiver voices in the development of self-management support tools.

## RESULTS

### Description of the study participants

Nine dyads (participant-caregiver pairs) completed two interviews, three to four months apart. All participants were men with a mean age of 65.3 (standard deviation = 3), and most were classified as GOLD Stage 4 (**Table 1**). All caregivers were women, with a mean age of 56.4 (standard deviation = 11), mainly spouses. Six of the nine dyads identified themselves as Malay.

**Table 1:** Participants and caregivers' characteristics

	Participants, n (%)	Caregivers, n (%)
**Total number**	9	9
**Sex**		
Male	9 (100.00)	0 (0.00)
Female	0 (0.00)	9 (100.00)
**Age, x̄ (SD)**	65.3 (3)	56.4 (11)
**Self-assigned ethnicity**		
Malay	6 (66.67)	6 (66.67)
Chinese	2 (22.22)	2 (22.22)
Indian	1 (11.11)	1 (11.11)
**COPD stages [** 2 ]*		
GOLD 3	3 (33.33)	
GOLD 4	6 (66.67)	
**Relationship with participant**		
Spouse		8 (88.89)
Daughter		1 (11.11)

COPD – chronic obstructive pulmonary disease, SD – standard deviation, GOLD – Global Initiative for Chronic Obstructive Lung Disease, x̄ – mean

*COPD is categorised into four stages based on the severity of airflow limitation, as measured by FEV1 (percentage of predicted): GOLD 1 (mild): ≥80% FEV1 predicted, GOLD 2 (moderate): 50–80% FEV1 predicted, GOLD 3 (severe): 30–50% FEV1 predicted, GOLD 4 (very severe): <30% FEV1 predicted.

Four main themes emerged from the analysis (**Table 2**).

**Table 2:** Summary of themes

	Sub-themes
**Theme 1: navigating economic constraints in COPD self-management**	Financial barriers limiting access to medical resources; innovative low-cost adaptations
**Theme 2: culturally embedded self-management: Integrating traditional and routine practices**	Embracing cultural practices; reframing daily chores as rehabilitation
**Theme 3: technology as a tool for home-based COPD care**	Participant-driven technological adaptations; absence of structured digital support
**Theme 4: family as partners in COPD management**	Mutual health benefits and dynamics; caregiver’s role in symptom management

COPD – chronic obstructive pulmonary disease

### Theme 1: navigating economic constraints in COPD self-management

#### Financial barriers limiting access to medical resources

**Figure 1:**
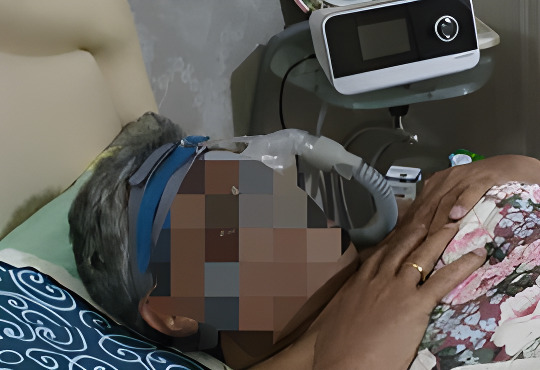
Sleeping with a continuous positive airway pressure machine. Source: provided by participants and captured using personal devices.

Participants described considerable economic challenges in managing COPD, despite generally good access to prescribed inhalers through the public health system. Financial barriers were most pronounced in accessing rehabilitative tools and essential supportive equipment, such as oxygen concentrators, continuous positive airway pressure machines, spirometry devices, and pulse oximeters. These were frequently out of reach due to cost, leading some to rely on temporary or loaned equipment while awaiting financial aid. A participant recalled (**Figure 1****)**:

‘My symptoms are better managed now with the new inhaler, but I need the machine (continuous positive airway pressure) to sleep, like the photo here. This one’s just on loan, which I have to pay. I’m eligible for support, but I’m still waiting for approval. It may take time.’ *– P4, 69-year-old man*

#### Innovative low-cost adaptations

**Figure 2:**
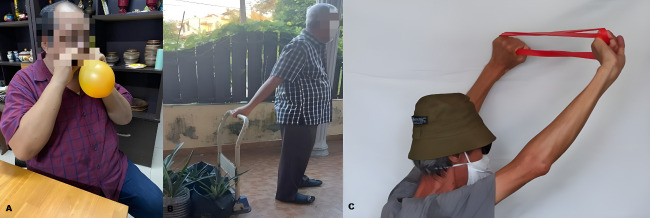
Home-based adaptations for rehabilitative tools. **Panel A.** Blowing a balloon. **Panel B.** Pulling a loaded trolley. **Panel C.** Exercising with a resistance band. Source: provided by participants and captured using personal devices.

In response to these limitations, participants demonstrated remarkable resourcefulness by developing low-cost, home-based adaptations for rehabilitative tools. Examples included using balloons for breathing exercises (**Figure 2**, Panel A) as an alternative to a formal breathing device, pulling loaded trolleys to mimic resistance training (**Figure 2**, Panel B) and resistance bands (**Figure 2**, Panel C**)** purchased from online marketplaces for strength training. A participant shared (**Figure 2**, Panel A):

‘I have no fancy machines at home, so I just blow into balloons; it’s cheap. This is the biggest I can manage!’ – P8, 59-year-old man

Participants saw these low-cost solutions as practical alternatives to conventional centre-based PR equipment, enabling sustained participation in self-management despite financial constraints. While these adaptations were valued, a few participants described them as ‘better than nothing’ but less effective than centre-based equipment, reflecting both ingenuity and a sense of compromise.

Beyond their practical function, these images symbolised resourcefulness and autonomy; balloons reflected participants’ ability to transform everyday, inexpensive objects into therapeutic tools, representing resilience and creativity in navigating financial constraints.

### Theme 2: culturally embedded self-management: Integrating traditional and routine practices

#### Embracing cultural practices

**Figure 3:**
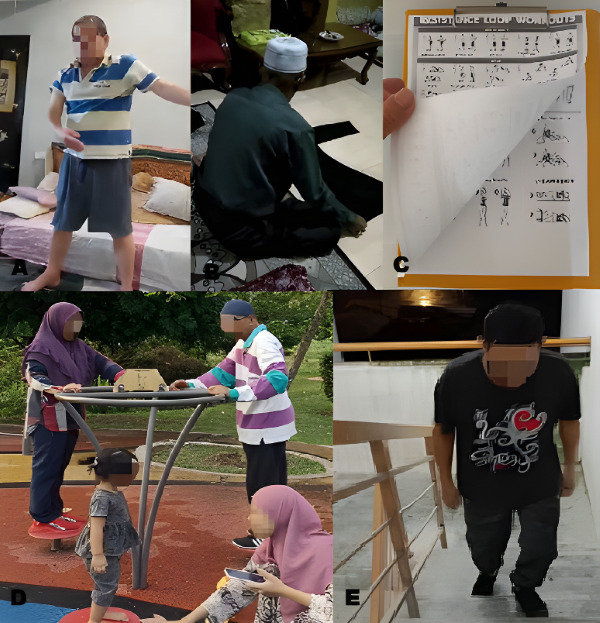
Activities at home. **Panel A.** Practising Qigong. **Panel B.** Folding clothes. **Panel C.** Referring to an exercise manual. **Panel D.** Exercising near home with family. **Panel E.** Going up and down the stairs. Source: provided by participants and captured using personal devices.

Cultural beliefs and practices were deeply woven into participants’ approaches to self-management. Rather than adopting a strictly biomedical framework, participants described COPD management as a holistic process that included physical, emotional, and spiritual dimensions. Traditional practices such as Chikung (Qigong) were widely adopted, with participants noting their calming and physically restorative effects. These exercises were not perceived as new but as extensions of familiar cultural routines, making them easier to integrate and sustain. Cultural familiarity increased acceptability and adherence, reflecting a preference for holistic approaches deeply rooted in local traditions. One participant explained (**Figure 3**, Panel A):

‘Doing Chikung helps me feel relaxed, and it feels natural because I grew up seeing elders doing it.’ *– P3, 64-year-old man*

In addition to movement-based practices, participants used culturally meaningful routines such as chanting and reading religious texts to manage anxiety and maintain psychological balance.

#### Reframing daily chores as rehabilitation

Many participants had limitations in physical activity, and they reframed daily domestic chores, such as folding clothes, sweeping floors, and hanging laundry to dry, as forms of exercise. These activities were perceived not only as productive but also as purposeful physical exertion that aligned with social expectations and household roles. This framing normalised activity, making it more acceptable and sustainable in the long term. A participant shared (**Figure 3**, Panel B):

‘I may not be able to do big exercises, but folding clothes like this keeps me moving. It’s part of my daily routine, and I see it as my form of exercise now.’ *– P7, 69-year-old man*

The integration of such culturally congruent practices enhanced participants’ motivation and reduced perceived barriers to self-management. Participants reported that these practices not only improved their physical condition but also restored a sense of dignity and control in the face of chronic illness.

Visually, images such as neatly folded laundry reinforced this narrative, conveying order and continuity and highlighting how self-management was embedded in valued social roles and daily rhythms, thereby affirming identity and self-worth despite illness.

### Theme 3: technology as a tool for home-based COPD care

#### Participant-driven technological adaptations

Participants widely used digital platforms to maintain continuity in their PR exercise after formal sessions ended. YouTube, Facebook, and TikTok were the most frequently mentioned sources of instructional content and manuals, with participants selecting and adapting exercises that matched their personal capacity and context. A participant said (**Figure 3**, Panel C):

‘Sometimes I watch exercise videos on Facebook. And these are simple exercises I found online (flipping the clipboard). I just picked what I could manage and kept going. Discipline is the hardest part, but I try to do it every day at home.’ *– P5, 65-year-old man*

These platforms enabled participants to maintain independence in their rehabilitation, offering flexible, on-demand access to exercises that could be practised safely at home. Some participants even modified online content into customised routines, demonstrating technological adaptability and self-efficacy. Photograph of printed online materials (**Figure 3**, Panel C) visually reinforced this adaptability, symbolising a blend of traditional and modern knowledge sources and reflecting a desire for personalised, trusted guidance amid the abundance of online content.

#### Absence of structured digital support

However, this independence also exposed a digital divide, not only in access but also in the ability to identify safe and effective content. While some participants customised their routines effectively, others relied on generic, unverified material. Several participants expressed a wish for more reliable, healthcare-validated resources, and caregivers raised concerns about misinformation, overexertion, and safety risks. A caregiver remarked:

‘My dad just scrolled for exercise on the internet, but whether these videos are appropriate for him, I’m not sure. Maybe it could be dangerous too, you know, like too much for him and potentially not good for his breathing.’ – *C2, 33-year-old woman*

In some dyads, such differences in perception led to disagreement, with caregivers discouraging certain online routines due to safety concerns and participants expressing frustration at these limits.

### Theme 4: family as partners in COPD management

#### Mutual health benefits and dynamics

Family involvement emerged prominently as a critical element in managing COPD. In many cases, caregiving was not unidirectional; instead, it was a shared health practice. Caregivers frequently attended rehabilitation sessions and exercised at or around home alongside participants, creating an environment of mutual encouragement and accountability. Images capturing these shared activities visually represented reciprocity, solidarity, and the blurring of conventional patient-caregiver boundaries, highlighting the relational nature of motivation and care. Both participants and caregivers noted reciprocal health benefits, with caregivers experiencing improvements in their own physical health and emotional resilience. A participant and caregiver jointly shared (**Figure 3**, Panel D):

‘Staying active helps me feel better, but what really keeps me going is my family: my wife, children, and grandchildren. They’re my reason to keep moving.’ – P9, 66-year-old man

‘It’s not just him, we exercise together. We remind and support each other, and that keeps both of us healthy.’ *– C9, 57-year-old woman*

However, some caregivers noted physical fatigue when balancing caregiving with other responsibilities, occasionally reducing their participation in shared activities and affecting the participant’s motivation.

#### Caregiver’s role in symptom management

Caregivers played essential roles in symptom monitoring, emergency response preparation, and maintaining participant motivation. The presence of family support significantly enhanced participants’ adherence to COPD self-management practices such as exercising, highlighting the importance of integrating caregiver involvement into COPD care programmes. A caregiver recalled (**Figure 3**, Panel E):

‘I always check his breathing before he starts going up and down the stairs. If he looks weak or the oxygen drops, I ask him to stop. We do this together, but I have to watch over him.’ – *C6, 52-year-old woman*

## DISCUSSION

### Summary of findings

In this study, we highlighted how people with COPD and their caregivers manage the disease in a low-resource setting. Financial barriers limited access to rehabilitation equipment, leading to creative, low-cost alternatives (*e.g.* balloons, resistance bands). Participants also drew on culturally meaningful practices such as Qigong and traditional breathing techniques to support physical and psychological well-being. Participants used digital platforms to sustain rehabilitation at home, though there was a call for validated, healthcare-curated content. Caregiving was reciprocal – while supporting participants, caregivers themselves experienced health benefits.

### Strengths and weaknesses

We used photo-elicitation methods to explore the lived experiences of daily activities in a low-resource setting, focussing on the often-overlooked caregiver roles. Photo-elicitation serves as both a narrative and reflective tool. It encourages participants and caregivers to document their everyday realities, exercise adaptations, and challenges, facilitating a more immersive and engaged form of data collection at two time points. One limitation of our study was the inability to follow participants across multiple time points, which could have provided insights into their experiences at different stages of disease progression. This limitation was primarily due to participants' unpredictable health conditions and limited research resources. However, to capture their perspectives within these constraints, we conducted interviews during and post-centre-based PR sessions. However, the requirement for participants to capture photographs excluded those without access to a mobile phone or camera, potentially skewing the sample towards more technologically literate or economically advantaged individuals.

We recruited participants and their caregivers from a single PR centre, which may limit the generalisability of the findings. At the time of data collection, this was the only public-funded centre in Selangor offering a structured PR programme. Expanding recruitment to multiple PR centres could increase the likelihood of including women and capture potentially different perspectives on lived experiences, which we were unable to do due to financial constraints. Nevertheless, the single-centre recruitment enabled the generation of rich, context-specific insights into COPD self-management and caregiving within a structured PR setting, which can inform similar services in comparable contexts. Additionally, we did not stratify experiences by comorbidity types or severity. While purposive sampling aimed to achieve demographic variation, all individuals attending PR during the recruitment period who met the inclusion criteria were men with COPD, reflecting the low prevalence of COPD among women in this setting [31]. This gender distribution reflects the epidemiology of COPD in this setting, rather than a selection bias. All caregivers were women, with roles that varied (*e.g.* daughters, wives), reinforcing the deeply ingrained societal expectations that position women as primary caregivers in this context. While we focussed on social and contextual dimensions of self-management, other components, such as medication adherence, exacerbation action plans, and smoking cessation, did not emerge as themes.

### Interpretation of results and comparison with the literature

A recent local study highlighted the urgent need for coordinated, compassionate care for people with very severe COPD in Malaysia, with a focus on the system-level and end-of-life gap [32]. In contrast, we complemented and extended this work by focussing on earlier phases of COPD self-management and uncovering actionable, patient- and caregiver-driven strategies. Using photo-elicitation dyadic interviews, findings suggest resourceful adaptations, such as low-cost tools, digital platforms, and culturally embedded practices, that support ongoing care in low-resource settings. These findings shift the focus from systemic shortcomings to community-based resilience, offering practical, upstream strategies that can strengthen long-term COPD management before palliative care becomes necessary.

Adaptive approaches, including the creative use of locally available substitutes, mirror findings from other LMICs where patients employ cost-saving strategies to preserve autonomy and functionality [15,33]. The integration of culturally rooted practices, such as Qigong, emphasises the importance of culturally congruent care models. These approaches have been linked to improved psychological well-being and self-efficacy, ultimately enhancing adherence to self-management [17,34]. Our findings reinforce global recommendations for aligning interventions with local belief systems to foster patient engagement.

Digital platforms were widely utilised to support home-based exercise, aligning with existing evidence on the utility of mobile health technologies for chronic disease management in settings with fragmented healthcare access [18]. However, variability in how participants engaged with these resources highlighted a digital divide in both access and digital health literacy. While some customised online routines worked effectively, others relied on generic, unverified content, raising concerns about safety and the spread of misinformation. Participants and caregivers expressed a need for structured, healthcare-endorsed digital resources, ideally co-designed with end users to ensure cultural relevance, safety, and sustained engagement. While previous studies have examined digital technologies and culturally adaptive practices separately [15,17,18,34], we illustrated how these elements can interact synergistically in daily COPD self-management, with culturally embedded practices serving as foundational rather than ancillary components of sustainable care routines.

Family caregiving emerged as a central pillar of long-term COPD self-management, aligning with evidence from Asian contexts where familial roles are integral to healthcare support [10,12]. Our findings highlight the reciprocal nature of caregiving within shared rehabilitation practices, positioning it not merely as a potential burden but as a source of mutual health reinforcement and emotional support. We offer an alternative perspective on caregiving, highlighting its potential to support positive behaviours across generations. By reframing caregiving as a dynamic and reciprocal health strategy, our findings emphasise the importance of involving families not only as support systems but as active co-beneficiaries in COPD self-management. This underexplored dimension of caregiving holds significant implications for global models of chronic disease care. Future interventions should incorporate structured, family-inclusive rehabilitation components that recognise and enhance these mutual benefits.

In the global context, the World Health Organization’s Package of Essential Noncommunicable Disease Interventions and ‘Best Buys’ frameworks prioritise clinical care, medication adherence, community engagement, and cost-effective interventions for LMICs [6,7]. While these recommendations are essential, they offer limited guidance on incorporating culturally grounded, low-cost adaptations into PR. Our findings suggest that global policy frameworks could be strengthened by explicitly recognising and promoting context-sensitive strategies. For example, substituting conventional PR equipment with locally available, affordable tools, integrating culturally familiar practices like Qigong to enhance adherence and self-efficacy, and embedding family-inclusive approaches that harness reciprocal caregiving benefits. Co-designing these strategies with patients, caregivers, and local communities can ensure cultural relevance, acceptability, and sustainability. Furthermore, formal guidance could incorporate the use of healthcare-curated digital resources as a scalable, low-cost means of supporting self-management in resource-limited settings. Incorporating these elements into global and national policy could bridge the gap between top-down recommendations and the lived realities of COPD management in LMICs.

### Implications for practice, research, and policy

Drawing from our findings, we prioritise three high-impact, feasible actions for immediate uptake by health policymakers, community-based organisations, and clinical teams in Malaysia and similar LMIC contexts:

1. Integrate low-cost, culturally resonant tools into PR to enhance sustainability and acceptability.
2. Formalise the use of digital health resources by developing healthcare-curated, vetted, and evidence-based content for patient and caregiver use, supported by training for healthcare providers to ensure safe and effective deployment.
3. Expand PR services into primary care and community settings to reduce access barriers, leveraging familial caregiving structures to extend programme reach.

Other essential but broader implications include fostering context-sensitive COPD care models, embedding culturally relevant practices within community rehabilitation, and combining these with validated, healthcare-curated digital tools to optimise patient engagement.

Future research should prioritise evaluating caregiver-mediated rehabilitation as a distinct model of care, assessing the long-term outcomes of culturally embedded and digitally supported self-management strategies, and examining the role of gender in caregiving.

## CONCLUSIONS

We highlight the resilience and creativity of participants and caregivers in managing COPD within constrained healthcare systems. Using photo-elicitation, we uncovered how cultural traditions, digital tools, and family relationships synergistically enable effective self-management. Effective COPD management in LMICs requires holistic strategies that go beyond clinical guidelines. Integrating culturally embedded practices, digital innovations, and reciprocal caregiving dynamics offers potentially scalable, contextually relevant models that can be adapted to similar low-resource settings, though further testing is needed. Policies informed by these insights may improve outcomes for chronic respiratory disease in similar contexts, fostering equitable and sustainable global health improvements.

## Additional material


Online Supplementary Document
