# Supplementary material for: Contextualising COPD self-management in Malaysia: insights from a qualitative photo-elicitation study of patients-caregiver dyads
Source: J Glob Health. 2025 Nov 7;15:04301. doi: 10.7189/jogh.15.04301 (PMC12599578; doi:10.7189/jogh.15.04301)
Supplement: Online Supplementary Document [file jogh-15-04301-s001.pdf]

Supplement to: Salim H, Fatin-Syazwani A, Zakaria N, Mohamad Yatim S, Chandrabose T, Ramdzan S, Chin Chan S, Mohamad F, Sazlina S. Contextualising COPD self-management in Malaysia: insights from a qualitative photo-elicitation study of patients-caregiver dyads. J Glob Health. 2015;15:04301.

Appendix S1. Summary of the centre-based PR programme

Overview:
The centre-based pulmonary rehabilitation programme is a multidisciplinary, evidence-based intervention primarily for individuals with moderate to severe Chronic Obstructive Pulmonary Disease (COPD). It aims to enhance quality of life, exercise tolerance, and self-management while reducing healthcare utilisation.

1. Objectives:
   - Improve health-related quality of life.
   - Promote independence in daily activities.
   - Enhance patient knowledge of COPD and treatment.
   - Improve exercise tolerance and muscle conditioning.
   - Reduce hospital admissions and length of stay over time.

2. Referral and Eligibility:
Referrals can be made by doctors, GPs, nurses, physiotherapists, or ward staff.
Inclusion criteria: Confirmed COPD diagnosis, FEV_1 <60%, recent CXR, functionally limited, stable and motivated.
Exclusion criteria: SpO_2 <90% at rest, unstable heart/lung disease, recent surgery, or other significant comorbidities affecting exercise.

3. Assessment
Initial assessment by PR nurse, physiotherapist, and occupational therapist includes:
   - CXR, spirometry, 6-minute walk test (6MWT), Borg Scale, quality of life & depression questionnaires.

Physician's medical review is required.

4. Programme delivery:
Sessions include a 1-hour exercise programme (individualised), warm-up, aerobic and resistance exercises (e.g., sit-to-stand, arm raises, leg extensions), and cool-down.
Education topics:
   - Lung anatomy & physiology
   - Breathing control techniques
   - Exacerbation management
   - Diet, energy conservation, and coping strategies

5. Team composition:
Includes a chest physician, respiratory nurse, physiotherapist, occupational therapist, dietician, pharmacist, counsellor, and medical social worker.
6. Venue Requirements:
Accessible space with parking, ground floor or lift access, facilities for shuttle walk, 10 exercise stations, and kitchen/toilets.
7. Follow-Up:
Conducted at 3 weeks and 3 months post-programme, using tools such as MRC Dyspnoea Scale, FEV_1, DASS, IADL, LCADL, DASI, 6MWT, CAT score, and Peak Cough Flow.

Appendix S2. Photo-taking prompts

- Capture a moment, place, or object that helps you feel at ease or makes breathing easier for you.
- Take a photo of an activity, exercises, or movements that is part of your daily routine or rehabilitation journey.
- Show a person, place, or thing that represents the support you receive in managing COPD. This could be a caregiver, a healthcare provider, or a meaningful object.
- Share a situation, space, or item that represents the difficulties you face when trying to stay active.
- Share an image that symbolises progress, motivation, or something that gives you hope in your COPD journey

Appendix S3. Semi-structured interview guide

Interview guide for participants	Interview guide for caregiver
Setting the scene<br>How are you feeling today?<br>Can you tell me about when your illness started, its management and what has changed since then?<br><br>On care, exercise and support<br>What type of care do you receive?<br>What do you know about exercise/keeping active for people living with COPD?<br>How do you feel about exercising in your current health state?<br>What types of exercise do you do at home?<br>What support do you receive?	Open questions<br>Can you tell me about your relationship with the patient?<br>How has your own health been affected since caring for the patient?<br>What kind of help and support does the patient need from you?<br>What have been the biggest challenges for you as a caregiver?<br><br>On care, exercise and support for the patient<br>What do you know about care and exercise for people living with COPD?

Suggestions for improvement<br>What would make life easier for you right now?<br>What advice would you give to others living with COPD?	What role do you play in helping the patient exercise/keep active?<br>What support have you received (or would like to receive) to help the patient manage at home?<br><br>Suggestions for improvement<br>What could improve the quality of life for both you and the patient?<br>What advice would you give to others caring for someone with COPD?

Appendix S4. Study rigour and trustworthiness

This study applied Lincoln and Guba's (1985, 1989) four criteria for trustworthiness, guided by the COREQ checklist to ensure methodological rigour.

1. Credibility

Member checking was conducted with two participant–caregiver dyads. This decision was informed by two factors:

1. Thematic saturation had been reached after analysis of the eighth dyad, with the ninth dyad confirming thematic stability, and
2. Resource constraints that limited the feasibility of repeating the process with all participants.

The selected dyads represented different COPD severities and cultural backgrounds. They were provided with a written summary of key findings in their preferred language and invited to comment on accuracy, resonance with their experiences, and any perceived omissions. Feedback indicated strong alignment between the summaries and their narratives, with only minor clarifications made.

2. Transferability

Thick description was achieved by providing detailed accounts of the study setting, participant characteristics, PR programme structure, and sociocultural context. This information allows readers to assess applicability to other contexts. Contextual details are summarised in the manuscript and expanded in Appendix S1 (PR programme description) and Appendix S2 (photo-taking prompts).

3. Dependability

An audit trail was maintained throughout the study, documenting recruitment processes, interview logistics, coding decisions, theme refinement, and reflexive notes. This audit

trail, stored in a secure shared drive, includes dated field notes, coding matrices, and meeting minutes from analytic discussions.

Triangulation occurred at several levels:

- Investigator triangulation: Four researchers (HS, AF, SS, SNR) independently coded the same transcripts and compared results to identify and resolve discrepancies.
- Data triangulation: Integration of textual interview data with visual data from photographs allowed multiple perspectives on the same phenomena, enriching interpretation and enhancing validity.
- Method triangulation: Using both dyadic interviews and photo-elicitation enabled exploration of relational dynamics as well as individual experiences.

4. Confirmability

Reflexive journaling (Details in Appendix S5) was maintained by the lead researcher (HS) throughout the study, capturing personal assumptions, evolving interpretations, and decisions made during data collection and analysis. Direct participant quotations and visual examples (where consented) were retained to provide a clear evidence trail linking findings to source material.

Appendix S5. Summary of the main investigator's reflexive journal: Navigating dual roles in research

Balancing my identity as a doctor and researcher

As a doctor, I am accustomed to diagnosing, advising, and offering solutions. However, stepping into the role of a qualitative researcher required a conscious shift. I had to listen without intervening, observe without directing, and allow participants to narrate their own experiences. At times, I found myself instinctively wanting to provide reassurance or medical guidance, particularly when participants described distressing symptoms. I had to remind myself that my role in this study was not to treat, but to understand.

Power dynamics: shaping participant responses

I became acutely aware of how my medical background influenced the way participants perceived me. Some viewed me as an authority figure, which may have influenced how openly they shared their struggles. A few patients hesitated when discussing dissatisfaction with their care, perhaps concerned about my professional affiliations. I made a deliberate effort to neutralise this dynamic; dressing casually, using open-ended questions, and emphasising that I was here to learn from their experiences, not to assess them.

Triangulating perspectives: patients and caregivers

Speaking to both patients and their caregivers provided invaluable insights. Patients often downplayed their limitations, expressing a desire to maintain independence. In contrast, caregivers described the daily struggles they witnessed: episodes of breathlessness, reluctance to accept help, and emotional challenges. This contrast reinforced the importance of considering multiple perspectives when understanding chronic illness. For instance, while one patient insisted, they were 'managing fine', their spouse later shared how they were concerned about trusting patients to independently managed themselves. These moments bring attention to the complexity of living with COPD, not just for patients but for their loved ones as well.

Emotional and ethical considerations

Hearing personal stories of frustration, loss, and resilience was deeply moving. There were moments when I felt a strong urge to offer comfort beyond my role as a researcher. One participant, for instance, expressed hopelessness over their declining physical abilities, saying, *'I used to be so active, but now I can't even walk without gasping for air.'* In these moments, I struggled with maintaining emotional distance. Instead of providing medical reassurance, I acknowledged their feelings and encouraged further reflection, allowing them to express their emotions freely.

Refining the research approach

As the study progressed, I adapted my approach based on my reflections. I became more mindful of how I framed questions especially prompts for the photographs, ensuring they encouraged deeper discussion rather than factual responses. I also took extra care in interviews with caregivers, acknowledging their emotional challenges while ensuring their voices were heard alongside those of the patients. Keeping this reflexive journal helped me remain aware of my biases, refine my interactions, and ensure that the study remained focused on capturing authentic lived experiences.

By continuously reflecting on these aspects, I aimed to uphold the integrity of the research, ensuring that participants' voices, not my own assumptions, shaped the findings.
